# Dr. Louis B. Pfandhoefer

**Published:** 1913-03

**Authors:** 


Dr. Louis B. Pfandhoefer, University of Buffalo, 1886, died at Falls City, Oregon, January 9, aged 60. His death was due to pneumonia.

